# Multidimensional Comparison of Microsurgical Clipping and Endovascular Techniques for Anterior Communicating Artery Aneurysms: Balancing Occlusion Rates and Periprocedural Risks

**DOI:** 10.3390/medicina61030498

**Published:** 2025-03-13

**Authors:** Vanessa Magdalena Swiatek, Amir Amini, Claudia Alexandra Dumitru, Lena Spitz, Klaus-Peter Stein, Sylvia Saalfeld, Ali Rashidi, I. Erol Sandalcioglu, Belal Neyazi

**Affiliations:** 1 Department of Neurosurgery, Otto-von-Guericke University, 39120 Magdeburg, Germany; vanessa.swiatek@med.ovgu.de (V.M.S.);; 2 Department of Simulation and Graphics, Otto-von-Guericke University, 39106 Magdeburg, Germany; 3 Research Campus STIMULATE, 39106 Magdeburg, Germany; 4 Department of Medical Informatics, University Hospital Schleswig-Holstein Campus Kiel, 24105 Kiel, Germany

**Keywords:** anterior communicating artery aneurysm, microsurgical clipping, endovascular treatment, intracranial aneurysm, ischemic complications, aneurysm occlusion

## Abstract

*Background and Objectives*: The anterior communicating artery is a common location for intracranial aneurysms. Anterior communicating artery aneurysms (AcomA) pose a significant risk of rupture. Treatment options include microsurgical clipping and endovascular techniques, but the optimal approach remains controversial. This study aims to compare the outcomes of these two treatment modalities in a single-center patient cohort using a comprehensive matching process based on clinical and morphological parameters. *Materials and Methods*: A retrospective analysis was conducted on 1026 patients with 1496 intracranial aneurysms treated between 2000 and 2018. After excluding cases lacking 3D angiography or aneurysms in other locations or without treatment, 140 AcomA were selected. The study matched 24 surgically treated AcomA cases with 116 endovascularly treated cases based on 21 morphological and clinical criteria, including age, sex, Hunt and Hess score, and Fisher grade. *Results*: The microsurgical clipping group demonstrated a significantly higher rate of complete aneurysm occlusion compared to the endovascular group (*p* = 0.007). However, this was associated with a higher incidence of postoperative ischemic complications in the surgical group (13 out of 24 cases) compared to the endovascular group (2 out of 116 cases). Despite these complications, no significant differences were found in clinical outcomes at discharge or follow-up, as measured by the modified Rankin Scale (*p* > 0.999). Both groups had comparable rates of hydrocephalus, vasospasm, and delayed cerebral ischemia. *Conclusions*: Microsurgical clipping resulted in higher aneurysm occlusion rates but carried an increased risk of ischemic complications compared to endovascular treatment. Clinical outcomes were comparable between the two modalities, suggesting that treatment decisions should be individualized based on aneurysm characteristics and patient factors. Further prospective studies are warranted to optimize treatment strategies for AcomA.

## 1. Introduction

The anterior communicating artery (Acom) is the most common site for intracranial aneurysms (IA), accounting for 30–37% of all cases [1,2,3]. Acom aneurysms (AcomA) carry a higher rupture risk compared to other IA types and are typically treated through surgical or endovascular methods [2,4,5,6,7,8,9,10]. Microsurgical treatment is complex due to the intricate anatomy and proximity to critical structures like the recurrent artery of Heubner and perforating arteries, requiring precise dissection to prevent complications such as intraoperative rupture or stroke [6,9,11]. While surgery was the preferred approach until the 1990s, the advent of endovascular techniques, particularly after the introduction of Guglielmi detachable coils [12], has shifted the treatment paradigm. Clinical trials, such as the International Subarachnoid Aneurysm Trial, showed better outcomes with endovascular treatment [13,14,15,16,17], though others, such as the Barrow Ruptured Aneurysm Trial, found no significant differences between surgical and endovascular approaches [18,19,20,21]. The optimal treatment strategy remains debated [2,21,22,23,24,25], with recent evidence suggesting that the choice may depend on aneurysm location and other factors [16,17,18,19,20,21].

Recently, Sattari et al. [3] conducted a comprehensive meta-analysis to evaluate the differences between surgical and endovascular treatments for AcomA. The analysis found no significant differences in mortality or poor functional outcomes between the two approaches, regardless of whether the aneurysms were ruptured or unruptured. However, surgical treatment demonstrated clear advantages, including significantly higher rates of aneurysm obliteration and lower rates of retreatment and recurrence compared to endovascular treatment. The risk of re-bleeding in ruptured aneurysms was similar between the two methods, and other clinical outcomes showed no substantial differences [3]. From a clinical perspective, it is well-recognized that more anatomically complex cases (wide neck or a small overall diameter of the aneurysm) are often preferentially assigned to surgical treatment, as it provides better visualization and access for managing intricate aneurysm anatomy, potentially influencing the previously stated results.

Moon et al. [26] demonstrated that advancements in adjunctive techniques and technology have made endovascular treatment a viable option for small or wide-necked ruptured AcomA, which were traditionally managed with surgical clipping. These aneurysms can now be effectively treated with endovascular embolization, without added risk or a higher likelihood of retreatment [26]. The largest comparative study on ruptured AcomA, conducted by Yang et al., additionally demonstrated that endovascular treatment offers superior clinical outcomes and comparable safety to surgical intervention, without an increased rate of retreatment [27]. However, this study did not account for aneurysm morphology, focusing solely on matching clinical parameters.

Our study aimed to explore these previous findings in a single-center cohort by comparing the surgical and endovascular treatment of AcomA. We utilized a multidimensional matching tool to compare 21 morphological and four clinical parameters between the two therapeutic strategies.

## 2. Materials and Methods

In this study, we conducted an initial screening of a retrospectively compiled database, which included 1026 patients diagnosed with 1469 saccular IA. These patients were treated at the Department of Neurosurgery, Otto-von-Guericke University Hospital, Magdeburg, Germany, between 2000 and 2018. The database was reviewed to identify cases that met the following inclusion criteria:

1. Availability of 3D angiographic imaging suitable for semi-automatic reconstruction of the aneurysm’s morphological features;
2. Aneurysm located at the Acom;
3. Treatment of the AcomA through either surgical or endovascular methods.

**Figure 1:**
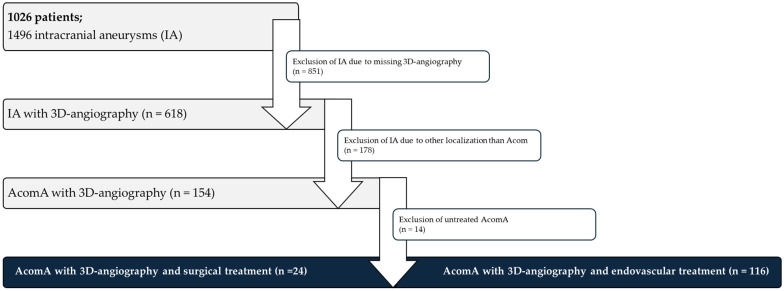
Flowchart depicting the patient selection process for the analysis of AcomA. A total of 1026 patients with 1496 IA were initially considered. Of these, 851 IA were excluded due to the absence of 3D angiography. Among the 618 IA with available imaging, 478 were excluded as they were not located at the Acom. Following further exclusion of 14 untreated AcomA, 140 treated AcomA remained for analysis. Of these, 24 underwent surgical intervention, while 116 received endovascular treatment.

The analysis of this retrospectively collected data were approved by the Ethics Committee of Otto-von-Guericke University (Ethics Vote Nr. RENOVA 94/20), with confirmation that all procedures conducted were part of standard clinical care. ChatGPT 4 by OpenAI was used for language editing. After the cohort was defined according to the inclusion criteria, 24 surgically treated AcomA cases were matched with 116 endovascularly treated AcomA cases based on relevant clinical variables. Furthermore, 21 semi-automatically extracted morphological parameters of the IA were incorporated into the matching process (Figure 1).

### 2.1. Data Acquisition

The clinical data for this study were retrospectively obtained through an extensive review of patients’ medical records, including anamneses, medication histories, and available diagnostic imaging. The collected parameters are outlined and defined as follows:

Epidemiological data:

- Age: defined as the patient’s age at the time of diagnosis
- Sex: classified as biological sex

Pre-existing conditions and risk factors:

- Hypertension: documented diagnosis or regular use of antihypertensive medication
- Nicotine abuse: history of or ongoing nicotine consumption
- Alcohol abuse: consumption exceeding 50 g of alcohol per week

Aneurysm-specific factors:

- Aneurysm rupture: determined based on intraoperative findings, imaging data, and CT evidence of hemorrhage patterns indicative of rupture
- Aneurysm multiplicity: defined as the presence of two or more IA

Clinical scores:

- Hunt and Hess score: a clinical assessment tool for the severity of subarachnoid hemorrhage (SAH) upon admission, ranging from minimal symptoms to deep coma and decerebrate posturing
- Fisher grade: a scale evaluating SAH severity based on CT findings, from no visible blood to substantial intracerebral or intraventricular hemorrhage
- Modified Rankin Scale (mRS) at discharge: a measure of neurological and functional disability at discharge, ranging from no symptoms to death

Treatment-related factors:

- Type of treatment:
  ○ Surgical approach (e.g., pterional craniotomy, anterior interhemispheric craniotomy)
  ○ Endovascular treatment method (e.g., single coiling, stent-assisted coiling)
- Number of procedures: total number of surgical or endovascular interventions performed
- Revisions: performance and type of revision surgery or intervention, if applicable

Complications associated with aneurysm therapy or rupture:

- Perioperative/peri-interventional ischemic complications: assessed through CT or MRI scans for evidence of ischemia
- Postoperative hemorrhage: assessed through postoperative CT scans
- Peri-interventional complications: including vessel occlusion or dissection during the procedure
- Hydrocephalus: symptomatic ventricular enlargement confirmed by CT imaging
- Hydrocephalus management: treatment involving placement of external ventricular drainage (EVD) or ventriculoperitoneal (VP) shunt
- Vasospasm: detected via transcranial Doppler ultrasound or angiography
- Vasospasm treatment: managed with endovascular spasmolysis or other endovascular techniques
- Delayed cerebral ischemia (DCI): characterized by new neurological deficits or impaired consciousness lasting over one hour, or the appearance of new ischemic changes or infarcts on imaging

Follow-up examinations:

- Follow-up duration: time period (in months) from initial patient admission to the latest follow-up
- mRS at follow-up: a reassessment of neurological and functional disability at follow-up, ranging from no symptoms to death

### 2.2. Morphological Analysis

**Figure 2:**
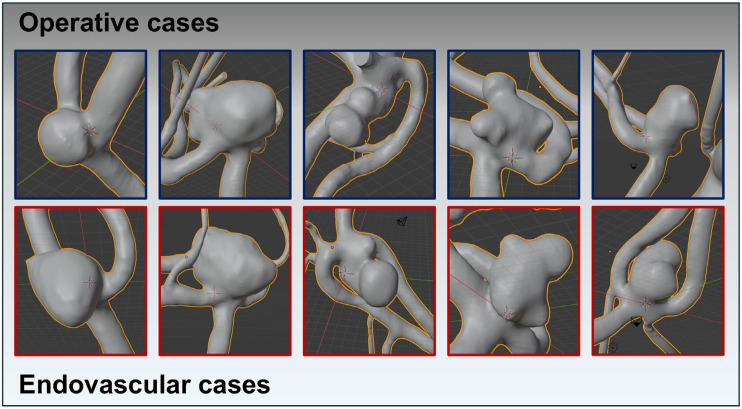
Using reprocessed and digitally subtracted 3D rotational angiography data, 3D surface models were generated following the technique outlined by Saalfeld et al. This figure exemplifies the success of the matching process, depicting very similar aneurysm morphologies across both treatment modalities. The upper row (squared in blue) shows the AcomA treated surgically, while the lower row (squared in red) illustrates the cases treated endovascularly.

Using the reprocessed and digitally subtracted 3D rotational angiography data, we generated 3D surface models following the technique outlined by Saalfeld et al. [28,29] (Figure 2). A semi-automatic segmentation of the aneurysm neck curve was then employed, enabling the automated extraction of the following 21 morphological parameters [28,29]:

- Hmax: maximum height of the aneurysm [28,30,31]
- Wmax: maximum width of the aneurysm perpendicular to Hmax [28]
- Dmax: maximum diameter of the aneurysm [28,31]
- Hortho: height of the aneurysm; measured vertically to the aneurysm neck [28,30,31]
- Wortho: maximum width of the aneurysm perpendicular to Hortho [28]
- Nmax: maximum diameter of the aneurysm neck [28,30,31]
- Navg: average diameter of the aneurysm neck [28,30,31]
- AR 1: aspect ratio 1; (Hortho/Nmax) [28,32,33]
- AR 2: aspect ratio 2; (Hortho/Navg) [28,32,33]
- EI: ellipticity index; (1 − 18^(1/3) V_CH^(2/3)/A_CH) [30,31]
- NSI: non-sphericity index; (1 − 18^(1/3) V^(2/3)/AA) [30,31]
- UI: undulation index; (1 − V/V_CH) [30,31]
- AA: surface of the aneurysm [30,31]
- OA 1: ostium area 1; surface of the aneurysm ostium [28]
- OA 2: ostium area 2; surface of the aneurysm ostium; the neck curve projected onto a plane [28]
- VA: volume of the aneurysm [30,31]
- V_CH: volume of the convex hull of the aneurysm [30,31]
- A_CH: surface of the convex hull of the aneurysm [30,31]
- Alpha: angle at point B1 describing the angle from the base line to the dome point [28]
- Beta: angle at point B2 describing the angle from the base line to the dome point [28]
- Gamma: angle at the aneurysm dome [28]

### 2.3. Matching Criteria and Cohort Matching

**Figure 3:**
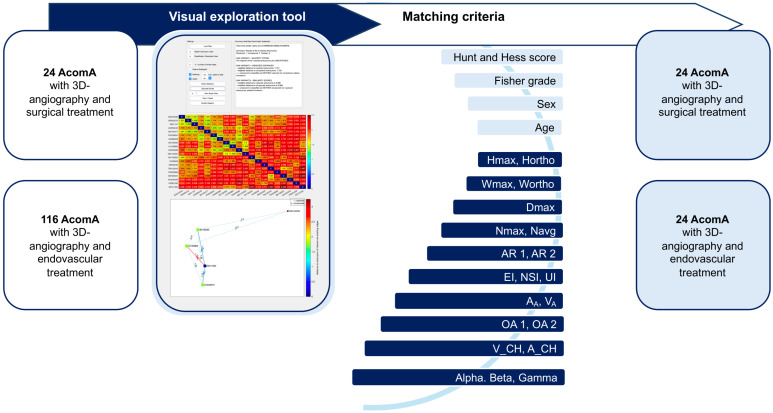
Flowchart illustrating the process of matching AcomA treated with either surgical or endovascular interventions using a visual exploration tool. A total of 24 AcomA treated surgically and 116 treated endovascularly were included. A matching process based on clinical and morphological criteria was employed. The matching criteria included the Hunt and Hess score, Fisher grade, sex, age, as well as 21 morphological parameters. This visual exploration tool enabled the systematic comparison and matching of aneurysms across the two treatment groups based on these detailed anatomical and clinical features.

To minimize the impact of confounding risk factors unrelated to the treatment modality and to ensure a balanced comparison of patient outcomes (including the functional outcome measured by mRS, the hydrocephalus and vasospasm rates, occlusion rates, and the incidence of stroke following IA treatment), we performed a systematic matching process. Each surgically treated AcomA was paired with the most comparable case treated endovascularly. A total of 24 surgically treated AcomA were matched and compared to a cohort of 116 AcomA treated through endovascular methods (Figure 3).

The matching process was based on a comprehensive set of parameters, including age at diagnosis, sex, and 21 morphological characteristics for unruptured AcomA cases, and additionally, the Hunt and Hess score and Fisher grade for ruptured AcomA cases. Given the substantial number of parameters involved, we employed an advanced interactive visual exploration tool, developed by Spitz et al. [34,35], to facilitate case-based reasoning for IA. Our research group has applied this matching approach in previous studies, where it is thoroughly explained [36,37]. Accordingly, we will provide only a short description below and highlight the specific elements relevant to the current analysis.

Data on the relevant parameters were input into an interactive visual exploration tool for case-based reasoning, which utilized a k-nearest neighbor (k-NN) classification to match surgically treated AcomA (aneurysm of interest, AOI) with endovascularly treated AcomA. The tool compared each AOI to a reference database and identified the five most similar cases (Figure 3).

Feature values were normalized using Z-score standardization, and dissimilarity between patients was calculated based on weighted feature differences. Three variants of the k-NN classifier were used: a simple classifier, one incorporating distance-based weighting, and another with distance normalization. Visual analytics techniques, such as summary panels and heat maps, were employed to explore the data interactively (Figure 3).

During the matching process, if the first identified match was unavailable due to prior assignment to another AOI, the next closest match was selected. Specifically, in four instances, the first match had already been assigned to another AOI (two unruptured and two ruptured cases). For the unruptured cases, the second most similar match was selected in both instances. In the ruptured cases, the third most similar match was chosen for one, and the fourth most similar match for the other. Ultimately, this matching procedure enabled a statistical comparison between 24 surgically treated AcomA and 24 endovascularly treated AcomA.

### 2.4. Statistical Analysis

Statistical analysis was performed using IBM SPSS Statistics, version 29. Categorical variables were analyzed using chi-square tests, with Fisher’s exact test used in cases where the expected frequency in any cell was less than five. For continuous or ordinal variables, the Kolmogorov–Smirnov test was used first to test for normal distribution, followed by Levene’s test to test for equality of variances.

In cases where the data were not normally distributed, the Mann–Whitney U test was used. For data with normal distribution, a *t*-test was used. The Bonferroni–Holm correction was applied to adjust for multiple comparisons.

## 3. Results

### 3.1. Cohort Overview

Before matching, the cohort included 140 patients, with 24 undergoing surgical treatment and 116 receiving endovascular intervention. Among the surgical group, 23 patients were treated via a pterional craniotomy, while one patient underwent an anterior interhemispheric approach. In the endovascular cohort, 20 patients were treated with single coiling, three with stent-assisted coiling, and one with an intrasaccular device. The mean age in the surgical group was 54.25 years, slightly higher than the 52.6 years in the endovascular group. Sex distribution was relatively balanced, with 38% male (9/24) and 62% female (15/24) in the surgical group, and 46% male (53/116) and 54% female (63/116) in the endovascular group (Table 1).

In terms of comorbidities, hypertension was present in 58% (14/24) of the surgical patients and 73% (85/116) of the endovascular patients. Nicotine abuse was documented in 54% of surgical patients and 52% of endovascular patients. Alcohol abuse was less common, affecting 13% of the surgical group and 15% of the endovascular group (Table 1).

Aneurysm rupture was present in 79% of both the surgical and endovascular groups. Multiple aneurysms were observed in 25% of the surgical patients and 24% of the endovascular patients. The mean follow-up duration was 22.5 months for the surgical group and 37.9 months for the endovascular group (Table 1).

In the matched cohort, hypertension was present in 58% (14/24) of the surgical patients and 63% (15/24) of those in the matched endovascular group. Nicotine abuse was observed in 54% (13/24) of the surgical group and 50% (12/24) of the endovascular group, with missing data reported for 8% and 12.5%, respectively. Alcohol abuse was documented in 13% (3/24) of patients in both groups, though there were more missing data in the surgical group (29% vs. 21%) (Table 1).

In terms of aneurysm characteristics, 79% (19/24) of patients in both groups presented with ruptured aneurysms. Aneurysm multiplicity was observed in 25% (6/24) of the surgical patients compared to 13% (3/24) in the matched endovascular group. When assessing clinical condition using the Hunt and Hess score in a cohort of 19 patients, the surgical group showed an even distribution across grades 1, 2, and 3, with 31.6% (6/19) in each grade, along with one patient (5.3%) in grade 5. By comparison, the matched endovascular group had 10.5% (2/19) of patients in grade 1, 47.4% (9/19) in grade 2, 36.8% (7/19) in grade 3, and 5.3% (1/19) in grade 4. For the Fisher grade, reflecting hemorrhage severity on CT scans, 5.3% (1/19) of surgical patients were classified as grade 1, 10.5% (2/19) as grade 2, 36.8% (7/19) as grade 3, and 47.4% (9/19) as grade 4. In the matched endovascular group, no patients were classified as grade 1 or 2, while 26.3% (5/19) were in grade 3, and 73.7% (14/19) were in grade 4 (Table 1).

Analysis of the morphological characteristics of all included AcomA, which were matched to the surgical cohort, revealed a predominance of complex configurations. The mean maximum diameter of the aneurysms was approximately 7 mm, with a mean neck width of about 5 mm. These characteristics suggest that the aneurysms in this study were generally large and with broad necks, which are known to influence treatment selection. Furthermore, the ellipticity index, non-sphericity index, and undulation index—which assess shape irregularity—were low, indicating that the aneurysms exhibited significant irregularity. This suggests that the included aneurysms had complex morphological features, such as non-spherical shapes and irregular contours, which are often associated with higher rupture risk and increased treatment difficulty.

Overall, the surgical and endovascular groups were well-matched in terms of age, sex, Hunt and Hess score, Fisher grade, and rupture status, although the endovascular group had a higher proportion of patients in the higher Fisher grades, particularly grade 3. After completing the matching process, the final cohort of 24 patients treated surgically and 24 patients treated endovascularly for AcomA was re-examined to verify the alignment of matching criteria between both groups. The quality of the matching was validated, with no significant differences found in the clinical parameters (age, sex, Hunt and Hess score, and Fisher grade) or the 21 morphological factors across the two cohorts (Table 2).

### 3.2. Analysis of Matched Cohort

**Figure 4:**
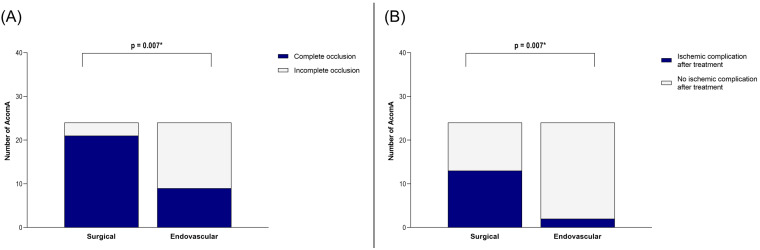
Illustration of the comparison between surgically and endovascularly treated AcomA regarding (**A**) occlusion rate and (**B**) occurrence of ischemic complications. * marks statistical significance.

The final analysis of the matched cohorts regarding clinical outcomes following surgical or endovascular treatment of AcomA aneurysms (assessed by the mRS at discharge and follow-up), the incidence of complications such as hydrocephalus and vasospasm, as well as associated re-interventions or delayed cerebral ischemia, and the occlusion rate and its correlation with post-procedural stroke occurrence yielded the following results:

Our analysis of the matched cohort revealed that the rate of complete occlusion was significantly higher in the surgical group compared to the endovascular group (*p* = 0.007) (Figure 4). However, this benefit came at the cost of an increased incidence of postoperative ischemic complications in the surgical group (*p* = 0.007) (Figure 4).

In the surgical group, ischemic complications were observed in 13 out of 24 cases. Five of these were attributed to vessel occlusions, while eight were likely caused by compression related to the surgical approach. In contrast, ischemic complications occurred in only two cases following endovascular treatment, both involving small embolic infarcts. Additional treatment-related complications were as follows: in the surgical group, intraoperative rupture of the AcomA occurred in eight cases (one incidental and seven SAH cases). Postoperative hemorrhage at the surgical site was detected in five cases, one of which required revision surgery. In the endovascular cohort, there was one case of transient middle cerebral artery occlusion, one procedure that had to be aborted, and one instance of common iliac artery dissection.

**Figure 5:**
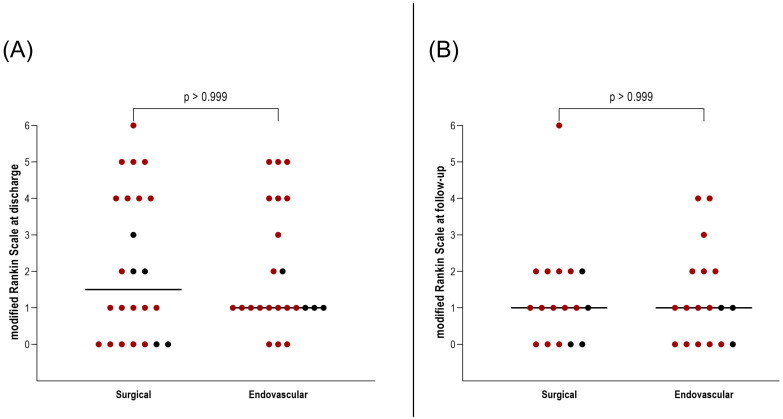
Illustration of the comparison between surgically and endovascularly treated AcomA regarding (**A**) mRS at discharge and (**B**) mRS at follow-up. The red-marked spots represent cases of SAH, while the black spots denote incidental cases.

**Figure 6:**
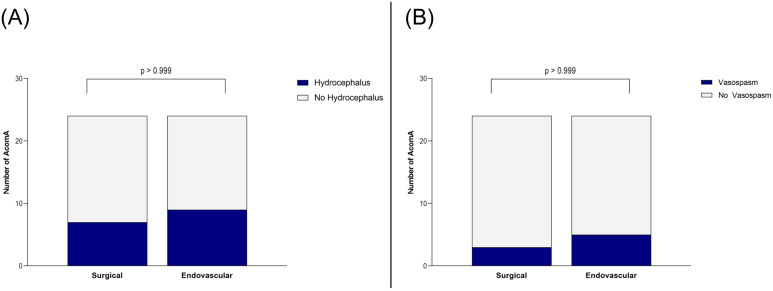
Illustration of the comparison between surgically and endovascularly treated AcomA regarding (**A**) occurrence of hydrocephalus and (**B**) of vasospasm.

Importantly, this did not translate into a worse clinical outcome. At discharge and during follow-up, no significant differences were found in mRS between patients who underwent surgical versus endovascular treatment for AcomA (*p* > 0.999) (Figure 5). Furthermore, the incidence of hydrocephalus and the need for EVD or ventriculoperitoneal shunt placement were comparable between the two groups (both *p* > 0.999) (Figure 6). Similarly, there were no differences in the rates of vasospasm or associated DCI (both *p* > 0.999) (Figure 6).

In cases of incomplete AcomA occlusion, one out of three incompletely treated cases in the surgical group underwent revision surgery, which successfully achieved complete occlusion. In the endovascular group, revision was performed in two out of fifteen cases with incomplete occlusion, both of which were also treated endovascularly and resulted in full occlusion. Of the remaining thirteen cases, one had no follow-up data, leaving the occlusion status unknown. In five cases, spontaneous occlusion of the residual perfusion occurred, ultimately leading to complete aneurysm occlusion. However, in seven cases, residual aneurysm perfusion persisted during follow-up.

## 4. Discussion

The comparison between microsurgical clipping and endovascular techniques for the treatment of AcomA remains a topic of ongoing debate, largely due to the distinct risks and clinical outcomes associated with each approach. In this study, we conducted a matching process, focusing on both the clinical aspects of patients and the morphological features of the AcomA.

In terms of aneurysm occlusion, previous studies have demonstrated that microsurgical clipping generally achieves higher immediate total occlusion rates compared to endovascular treatment. The meta-analysis by Diana et al. highlights a post-treatment occlusion rate of 98.2% for microsurgical clipping, which is significantly higher than the 87.1% seen with stent-assisted coiling and the 47.6% observed with endosaccular devices [38,39]. These findings are in line with Sattari et al., who reported that surgical clipping results in a significantly higher rate of aneurysm obliteration compared to endovascular techniques [3]. This suggests that, while endovascular treatments are increasingly used and continue to improve, microsurgical clipping may still be more reliable for achieving immediate aneurysm obliteration, particularly in complex anatomical cases of AcomA. Our analysis of the matched cohort supports these findings, revealing that the rate of complete occlusion was significantly higher in the surgical group compared to the endovascular group.

However, the higher occlusion rates associated with microsurgical clipping come at a cost. Previous studies indicate that microsurgical clipping is linked to a higher rate of major complications. Diana et al. reported a 7.1% major complication rate with clipping, compared to 4.4% for stent-assisted coiling, although these differences were not statistically significant [38]. Yang et al. strengthened these findings, noting that microsurgical clipping was associated with higher incidences of intraoperative rupture, hydrocephalus, and ischemic events, while endovascular treatment, particularly stent-assisted coiling, tend to have fewer procedure-related complications [27]. In our analysis, an increased incidence of postoperative ischemic complications in the surgical group with 13 out of 24 cases was observed. Five of these were attributed to vessel occlusions, while eight were likely caused by compression related to the surgical approach. In contrast, ischemic complications occurred in only two cases following endovascular treatment, both involving small embolic infarcts. Additional treatment-related complications in the surgical group included intraoperative rupture of the AcomA in eight cases (one incidental and seven SAH cases). We are aware that the intraoperative rupture rate in this selective cohort appears unusually high. Therefore, we analyzed our institution’s data regarding intraoperative ruptures during aneurysm clipping before and after the establishment of the specialized neurovascular center in 2019, as the presented cohort was treated prior to this time. Due to the retrospective nature of this study, we do not have an exact algorithm detailing how the decision to treat an aneurysm surgically or endovascularly was made. However, the decision was always reached in an interdisciplinary manner by the treating neuroradiologist and neurosurgeon. This process inherently introduces a selection bias, as the treatment modality may have been influenced by the attending physician on duty at the time. This is reflected in our cohort, where even some anatomically complex AcomA with wide necks were treated endovascularly, despite the fact that, under more objective evaluation, microsurgical clipping may have been the preferable choice. In some cases, clear indications favoring surgical treatment, such as a significant intracerebral hemorrhage component, or contraindications against surgery, such as severe coagulopathy, were not always strictly considered in the decision-making process. This variability may have had an impact on treatment outcomes. It should also be noted that, prior to the establishment of the neurovascular center, there was a tendency to favor endovascular treatment for AcomA. This selection bias is reflected in the present study, where the surgically treated AcomA tended to be more complex in configuration. The higher anatomical complexity of these aneurysms likely contributed to the observed rate of intraoperative rupture in the pre-central cohort.

With the introduction of the specialized neurovascular center, structural changes in surgical techniques aimed at preventing intraoperative ruptures were implemented and strictly enforced. These changes included refined dissection techniques, improved preoperative imaging, and enhanced intraoperative monitoring, all contributing to the reduction in rupture rates. Furthermore, from that point onward, all patients initially underwent a cranial CT scan with CT angiography, followed by digital subtraction angiography within 12 h. The decision for either microsurgical clipping or endovascular treatment was made in an interdisciplinary manner by a specialized neurovascular surgeon and a specialized neuroradiologist. Treatment was subsequently performed using the chosen modality, and all patients were monitored in the intensive care unit for at least 14 days. During this period, daily transcranial Doppler examinations were conducted to assess for vasospasm. If the transcranial Doppler was not feasible due to poor quality, regular CT angiography with perfusion imaging was performed instead. In cases of hydrocephalus, EVD was placed when necessary. To optimize cerebral perfusion, the mean arterial pressure was maintained above 100 mmHg starting from the third day after SAH and after aneurysm treatment. Complete aneurysm occlusion was confirmed initially via digital subtraction angiography and subsequently monitored using MRI during follow-up.

The data revealed that, before 2019, the intraoperative rupture rate was 18%, which is considered high. However, following the establishment of the specialized neurovascular center in 2019, the rupture rate was reduced to 9%, with all cases of intraoperative rupture involving previously ruptured aneurysms with SAH. Furthermore, it should be noted that the cohort presented in this study, due to the strict inclusion criteria, predominantly included morphologically complex and large AcomA. This complexity likely contributed to the higher incidence of intraoperative ruptures, as larger and more intricate aneurysms are inherently associated with increased technical challenges during microsurgical clipping. Postoperative hemorrhage occurred in five cases, one of which required revision surgery. In the endovascular cohort, there was one case of transient middle cerebral artery occlusion, one procedure that had to be aborted, and one instance of common iliac artery dissection. Despite these differences, the incidence of hydrocephalus and the need for EVD or ventriculoperitoneal shunt placement were similar between the two groups. There were also no differences in vasospasm rates or associated DCI.

Importantly, the higher rate of ischemic complications did not translate into worse clinical outcomes. At discharge and follow-up, no significant differences in mRS scores were found between patients who underwent surgical versus endovascular treatment for AcomA aneurysms. However, these findings diverge from some previously published results. While Sattari et al.’s meta-analysis showed similar risks to ours [3], other studies suggest that endovascular treatment tends to yield better clinical outcomes. For instance, Diana et al. reported that good clinical outcomes were significantly higher in the endovascular treatment groups compared to microsurgical clipping at 12 months [38]. Similarly, a two-year follow-up study by Yang et al. showed that endovascular treatment patients had better functional independence and lower mortality rates compared to those who underwent microsurgical clipping [27].

In our cohort, one of three surgically treated cases with incomplete AcomA occlusion underwent successful revision surgery. In the endovascular group, two of fifteen cases had revision, both leading to full occlusion. Of the remaining thirteen, one had no follow-up data, five achieved spontaneous occlusion, and seven had persistent residual perfusion leading to a higher rate of not occluded AcomA in endovascular treated cases. In line with these results, Diana et al. and Sattari et al. both indicate that microsurgical clipping leads to lower retreatment and recurrence rates compared to endovascular treatment [3,38].

This study has several limitations. First, it is a retrospective, single-center analysis, which may introduce selection bias and limit the generalizability of our findings. While we employed a matching process to reduce confounding variables, certain factors, such as surgeon expertise or patient-specific anatomical variations, may still have influenced the outcomes. Additionally, although aneurysm size and location were matched, comorbidities were not included in the matching process. Instead, we prioritized the initial severity of the SAH based on the Hunt and Hess score and Fisher grade, as the limited cohort size restricted the number of parameters that could be included without reducing the sample further. The relatively small sample size, particularly in the surgical cohort, also limits the statistical power to detect differences in less common complications or outcomes. Finally, although we incorporated a wide range of clinical and morphological parameters in the matching process, other potential confounders, such as the influence of hospital protocols or technological advancements over time, were not fully accounted for.

To enhance the generalizability and applicability of these findings, future research should focus on conducting multicenter prospective studies with larger patient cohorts and standardized treatment protocols. A broader dataset incorporating multiple institutions would allow for a more comprehensive assessment of factors influencing treatment selection and outcomes. Such studies would provide more robust evidence to guide treatment decision-making and optimize management strategies for AcomA.

## 5. Conclusions

In summary, microsurgical clipping for AcomA achieved a higher rate of complete occlusion but was associated with an increased risk of ischemic complications compared to endovascular treatment. Despite the higher incidence of these complications, clinical outcomes, as measured by the mRS, were not significantly worse. Additionally, there were no differences between the surgical and endovascular groups in other complications such as hydrocephalus or vasospasm. Endovascular techniques, however, showed higher rates of residual perfusion and a greater likelihood of requiring retreatment. The choice of treatment modality should be individualized, taking into account both the aneurysm’s complexity and the patient’s overall risk profile. Further prospective multicenter studies are necessary to validate these findings and optimize treatment strategies for AcomA.

## Figures and Tables

**Table 1:** Comparison of age, sex, comorbidities (hypertension, nicotine, and alcohol abuse), aneurysm characteristics (rupture status, multiplicity), follow-up duration, and clinical grading (Hunt and Hess scale and Fisher grade) between the surgical, matched endovascular, and whole endovascular cohorts.

							Whole Cohort						Matched Cohort					
	Surgical (n = 24)						Endovascular (n = 116)						Endovascular (n = 24)					
Age (mean)	54.25 years						52.6 years						51.8 years					
Sex	9 male; 15 female						53 male; 63 female						9 male; 15 female					
	yes		no		n.i.		yes		no		n.i.		yes		no		n.i.	
Hypertension	14		10		0/0%		85		31		0/0%		15		9		0/0%	
Nicotine abuse	13		9		2/8.3%		60		30		26		12		9		3/12.5%	
Alcohol abuse	3		14		7/29.2%		17		69		30		3		16		5/20.8%	
Rupture status	19		5		0/0%		92		24		0/0%		19		5		0/0%	
Multiplicity	6		18		0/0%		28		86		2		3		21		0/0%	
Follow-up duration	22.5 months				0/0%		37.9 months				0/0%		39.6 months				0/0%	
	1	2	3	4	5	n.i.	1	2	3	4	5	n.i.	1	2	3	4	5	n.i.
Hunt and Hess score	6	6	6	0	1	0	7	38	25	14	7	1	2	9	7	1	0	0
	1	2	3	4	n.i.		1	2	3	4	n.i.		1	2	3	4	n.i.	
Fisher grade	1	2	7	9	0		2	2	28	59	1		0	0	5	14	0	

n.i. = no information.

**Table 2:** Post-matching comparison of clinical and morphological parameters between surgical and endovascular treated AcomA, confirming balanced matching with no significant differences in key factors.

	Surgical (n = 24)	Endovascular (n = 24)	Statistical Analysis
Age (mean)	54.25 years	51.8 years	*p* > 0.999 *
Sex	9 male; 15 female	9 male; 15 female	*p* > 0.999 ***
Hunt and Hess score (median)	2	2	*p* > 0.999 **
Fisher grade (median)	3	4	*p* > 0.999 **
Hmax (mean)	6.2	5.8	*p* > 0.999 *
Wmax (mean)	6.3	6.0	*p* > 0.999 *
Dmax (mean)	8.1	7.4	*p* > 0.999 *
Hortho (mean)	5.4	5.4	*p* > 0.999 *
Wortho (mean)	7.3	6.4	*p* > 0.999 *
Nmax (mean)	5.0	4.8	*p* > 0.999 *
Navg (mean)	4.2	4.2	*p* > 0.999 *
AR 1 (mean)	1.1	1.2	*p* > 0.999 *
AR 2 (mean)	1.3	1.3	*p* > 0.999 *
EI (mean)	0.3	0.3	*p* > 0.999 *
NSI (mean)	0.2	0.2	*p* > 0.999 *
UI (mean)	0.1	0.1	*p* > 0.999 *
A_A_ (mean)	113.8	101.6	*p* > 0.999 **
OA 1 (mean)	15.7	15.8	*p* > 0.999 *
OA 2 (mean)	14.0	13.9	*p* > 0.999 *
V_A_ (mean)	126.7	110.8	*p* > 0.999 **
V_CH (mean)	148.2	126.8	*p* > 0.999 **
A_CH (mean)	133.1	119.5	*p* > 0.999 **
Alpha (mean)	77.7	78.4	*p* > 0.999 **
Beta (mean)	63.7	61.3	*p* > 0.999 **
Gamma (mean)	38.6	40.2	*p* > 0.999 *

*t*-test with Bonferroni–Holm correction *; Mann–Whitney U test with Bonferroni–Holm correction **; chi-square test with Bonferroni–Holm correction ***.

## Data Availability

The datasets obtained and analyzed during the current study are available from the corresponding author on reasonable request.

## Abbreviations

The following abbreviations are used in this manuscript:

Acom	Anterior communicating artery
AcomA	Anterior communicating artery aneurysm
AOI	Aneurysm of interest
DCI	Delayed cerebral ischemia
EVD	External ventricular drainage
IA	Intracranial aneurysms
mRS	Modified Rankin scale
SAH	Subarachnoid hemorrhage
VP	Ventriculoperitoneal
